# Improving respiratory muscle strength in patients with multiple sclerosis through respiratory muscle training: a systematic review and meta-analysis

**DOI:** 10.7717/peerj.20876

**Published:** 2026-04-09

**Authors:** Tianyu Chen, Yu Jin, Nan Jiang, Junyue Lu, Meng Wei, Haoyuan Huang, Youliang Wen

**Affiliations:** 1 Rehabilitation College, Gannan Medical University, Ganzhou, Jiangxi Province, China; 2 Rehabilitation College, Pingliang Vocational and Technical College, Pingliang, Gansu Province, China

**Keywords:** Multiple sclerosis, Respiratory muscle training

## Abstract

**Introduction:**

Multiple sclerosis (MS) has a high incidence and can occur at all ages, and respiratory dysfunction is a leading cause of death among the complications of MS. Respiratory muscle training (RMT) is often used to help MS patients improve their respiratory function, but the specific impact of RMT has not been clearly elucidated. The present meta-analysis aims to evaluate the impact of RMT on MS patients.

**Methods:**

We looked up PubMed, Cochrane Library, Embase, Web of Science, and PEDro with the query “respiratory muscle training” AND “multiple sclerosis”. The cutoff was January 6, 2026. After screening, eligible randomized controlled clinical trials were analyzed to calculate the standardized mean differences (SMDs) and 95% confidence intervals (CI) of the following metrics regarding RMT intervention: maximal inspiratory pressure (MIP), maximal expiratory pressure (MEP), forced vital capacity (FVC), forced expiratory volume in one second (FEV_1_), and the ratio of forced expiratory volume in one second to forced vital capacity (FEV_1_/FVC).

**Results:**

A total of 370 articles were retrieved, and eight remained after rigorous screening. The eight trials included a total of 249 patients. Patients undergoing RMT exhibited significant improvements in MEP (SMD = 0.42, 95% CI [0.09–0.76], *P* = 0.01, I^2^ = 24%), MIP (SMD = 0.32, 95% CI [0.02–0.63], *P* = 0.04, I^2^ = 17%), FEV_1_ (SMD = 0.41, 95% CI [0.08–0.74], *P* = 0.01, I^2^ =9%), and FEV_1_/FVC (SMD = 0.52, 95% CI [0.15–0.89], *P* = 0.005, I^2^ =0%), but FVC did not benefit from RMT (SMD = 0.28, *P* = 0.06).

**Conclusions:**

Respiratory muscle training can improve respiratory muscle strength and the lung function in MS patients.

## Introduction

Multiple sclerosis (MS) (Yamout & Alroughani, 2018) is a chronic autoimmune disease characterized by the demyelination and neurodegeneration of the central nervous system (McGinley, Goldschmidt & Rae-Grant, 2021; Doshi & Chataway, 2016). It is known for its potential to cause significant disability over time. This progression is marked by specific functional impairments, such as compromised motor control, sensory deficits, and fatigue, which collectively limit a person’s ability to perform daily activities and participate in life roles, ultimately leading to significant disability (Marcus, 2022; Garg & Smith, 2015). The disease is attributed to the disruption of the ascending sensory and descending motor pathways of the central nervous system, and the peripheral nerves are typically not directly affected (He, Guo & Rao, 2023). Because of the disruption, the brain attempts to reorganize its neural networks—a process known as neuroplasticity (Rahnemayan et al., 2025)—to compensate for impaired communication, and such reorganization may alter the function, shape, and structure of the brain, reflecting both adaptive and, at times, maladaptive changes. However, despite these compensatory efforts, patients often experience persistent functional deficits not only in areas directly impacted by the lesions but also in regions dependent on intact neural connections below the level of the injury. The disease can occur at any age, though its onset most commonly falls between 20 and 40 years old. Additionally, it is more prevalent in women than in men, with reported female-to-male ratios typically ranging from about 2:1 to 3:1 (Koch-Henriksen & Magyari, 2021). MS poses a substantial global health burden, affecting approximately 2.3 million people worldwide (Haki et al., 2024) and nearly 1 million people in the United States alone (Wallin et al., 2019). Furthermore, the epidemiology of MS reveals a distinct geographical gradient, with prevalence generally increasing with distance from the equator. Populations of Northern European descent exhibit a higher risk, though rising incidence in regions like the Middle East (*e.g.*, a reported prevalence of 4.4/100,000 in Iraq) highlights the complex interplay of genetic predisposition and environmental factors in its etiology (Haki et al., 2024).

The clinical manifestations of MS is high diverse, encompassing significant fatigue, vision problems such as blurred vision or optic neuritis, difficulty in walking or maintaining balance, and numbness or weakness (especially in the limbs). They may also have cognitive impairment, muscle stiffness (spasticity), depressive disorders, and urinary difficulties (Marcus, 2022; Samjoo et al., 2023; Katz Sand, 2015). Among these different complications, ventilatory dysfunction (the impaired mechanical movement of air) represents a critical concern, as it directly leads to impaired respiration (gas exchange) and is associated with mortality (Carter & Noseworthy, 1994). Data indicate that 40% of patients with MS have respiratory muscle weakness (Ward & Goldman, 2022). The pathophysiology of ventilatory dysfunction in MS is primarily attributed to the demyelination of corticospinal tracts that innervate the respiratory muscles, including the diaphragm, intercostals, and ventilatory muscles. This disrupts the efficient neural drive from the respiratory centers in the brainstem to the muscle fibers, leading to reduced maximal force generation, impaired coordination, and decreased endurance (Haki et al., 2024). This ventilatory impairment stems from weakened ventilatory muscles in MS patients (Halabchi et al., 2017; Jørgensen et al., 2017; Bianchini et al., 2020), which compromises cough efficacy and can lead to life-threatening aspiration pneumonia and acute respiratory failure (Kimoff, Kaminska & Trojan, 2022; Jones, Enright & Busse, 2012).

The weakness of respiratory muscles is characterized by reduced force generation, spastic hypertonicity, and progressive muscle atrophy (Wei, 2014; Owens, 2016). Respiratory muscle training (RMT) is a targeted therapeutic modality to mitigate ventilatory muscles weakness by enhancing neuromuscular activation, thus effectively improving ventilatory function and cough ability (Rietberg et al., 2017). Through inspiratory muscle training (IMT) and/or expiratory muscle training (EMT) (Göhl et al., 2016), RMT not only promotes hypertrophy—as evidenced by an increase in the cross-sectional area of muscle fibers—but also enhances muscle endurance and coordination (Mackała et al., 2019), thus leading to clinically measurable improvements in oxygen uptake, delivery, and overall respiratory function. In addition to exercising the breathing muscles, RMT also involves adjusting the breathing pattern (Pardy, Reid & Belman, 1988), with a focus on controlling the rhythm, depth, *etc.* (Samjoo et al., 2023). Clinicians now increasingly apply RMT as an adjunct therapy for respiratory rehabilitation (Saxby et al., 2024; Grubic Kezele et al., 2023; Grubić Kezele et al., 2020; Ghannadi et al., 2022; Abasıyanık et al., 2020). For patients of neurological diseases such as spinal cord injury and stroke, RMT has been proven to improve respiratory function, reduce the occurrence of complications such as acute respiratory deficiency, atelectasis, or aspiration pneumonia (Carter & Noseworthy, 1994), and increase the patients’ quality of life (Pollock et al., 2013; Watson et al., 2022). For patients with chronic obstructive pulmonary disease, RMT also improves the ability to ventilate (Ammous et al., 2023; Beaumont et al., 2015). Ferreira et al. (2016) suggested that RMT can serve as an adjunct therapy in neurodegenerative disease rehabilitation to improve both ventilation function and respiratory intensity. Ventilation function refers to the lungs’ capacity to move gas per unit time, a key indicator of respiratory mechanical efficiency, while respiratory intensity reflects the work performed by respiratory muscles to meet the metabolic demands of breathing.

In recent years, many studies have been reported on the effects of RMT on MS patients, as it helps in managing fatigue and other MS symptoms that can be attributed to weak respiratory muscles. For example, Rietberg et al. (2017) demonstrated that RMT significantly improves the maximum inspiratory pressure (MIP) in MS patients (Westerdahl et al., 2021). However, the efficacy of RMT on the ventilatory function of MS patients has not been summarized systematically. Therefore, the present meta-analysis aims to analyze the effects of RMT on the ventilatory function of MS patients.

## Methods

### Protocol and registration

This systematic review was reported according to the PRISMA guidelines and the Cochrane Handbook for Systematic Reviews of Interventions (Page et al., 2021). All analysis methods were documented on the PROSPERO platform with the registration number CRD42024525413.

### Literature search and selection

We searched PubMed, Embase, PEDro, Web of Science, and Cochrane Library with the following query: “respiratory muscle training” AND “multiple sclerosis” (Page et al., 2021). The time frame was from the inception of the database to January 6, 2026. TC and YJ independently conducted the searches. The records were screened simultaneously based on the inclusion and exclusion criteria. All the differences arising during this process were analyzed by the corresponding author (YW), and everyone discussed together to reach a unanimous conclusion.

The articles were included if (1) the patients were diagnosed with MS, (2) the intervention group willingly underwent various forms of RMT, (3) the study design adhered to a randomized controlled trial (RCT) framework, and (4) the measurement results included the ratio of forced expiratory volume in one second to forced vital capacity (FEV_1_/FVC), forced expiratory volume in one second (FEV_1_), maximal inspiratory pressure (MIP), maximal expiratory pressure (MEP), and/or forced vital capacity (FVC), or any of them. The articles were excluded if (1) there were insufficient data points meeting the filter criteria for analysis, (2) the trial protocol was not described clearly, or (3) registration in a trial registry was lacking.

### Data extraction and validation

Data were extracted from the studies that met the inclusion criteria by two authors. A data extraction table was created to capture the following information: author(s), publication year, trial location, literature type, patient demographics including age, Expanded Disability Status Scale (EDSS) score, and disease subtype for both intervention and control groups, intervention methods, and intervention outcomes. The extracted data were crosschecked and validated by other authors. The final confirmation of the master table was made after a thorough discussion and with consensus from all authors.

### Quality assessment

Two authors read the full text of the included literature and assessed the risk of bias independently and objectively using Review Manager 5.3. The assessment involved the following aspects: (1) random sequence generation (selection bias), (2) allocation concealment (selection bias), (3) blinding of participants and personnel (performance bias), (4) blinding of outcome assessment (detection bias), (5) incomplete outcome data (attrition bias), (6) selective reporting (reporting bias), and (7) other bias. For each of the above, the risk of bias was assigned as low, unclear, or high. Any disagreement was resolved after a discussion to attain consensus.

### Data analysis

All analyses were carried out using Review Manager 5.3. A random effects model was applied. Effect size was used to evaluate the difference between the RMT group and the control group (*i.e.,* difference of the standard deviation and the mean). Statistical heterogeneity was assessed using Cochran’s Q test and the inconsistency test. Heterogeneity was categorized as follows: I^2^ < 25%, low; 25% ≤ I^2^ < 50%, moderate; 50% ≤ I^2^ < 75%, substantial; I^2^ > 75%, high. Differences were considered statistically significant if *P* < 0.05 and the effect estimate fell within the 95% confidence interval (95% CI).

## Results

### Study selection

**Figure 1:**
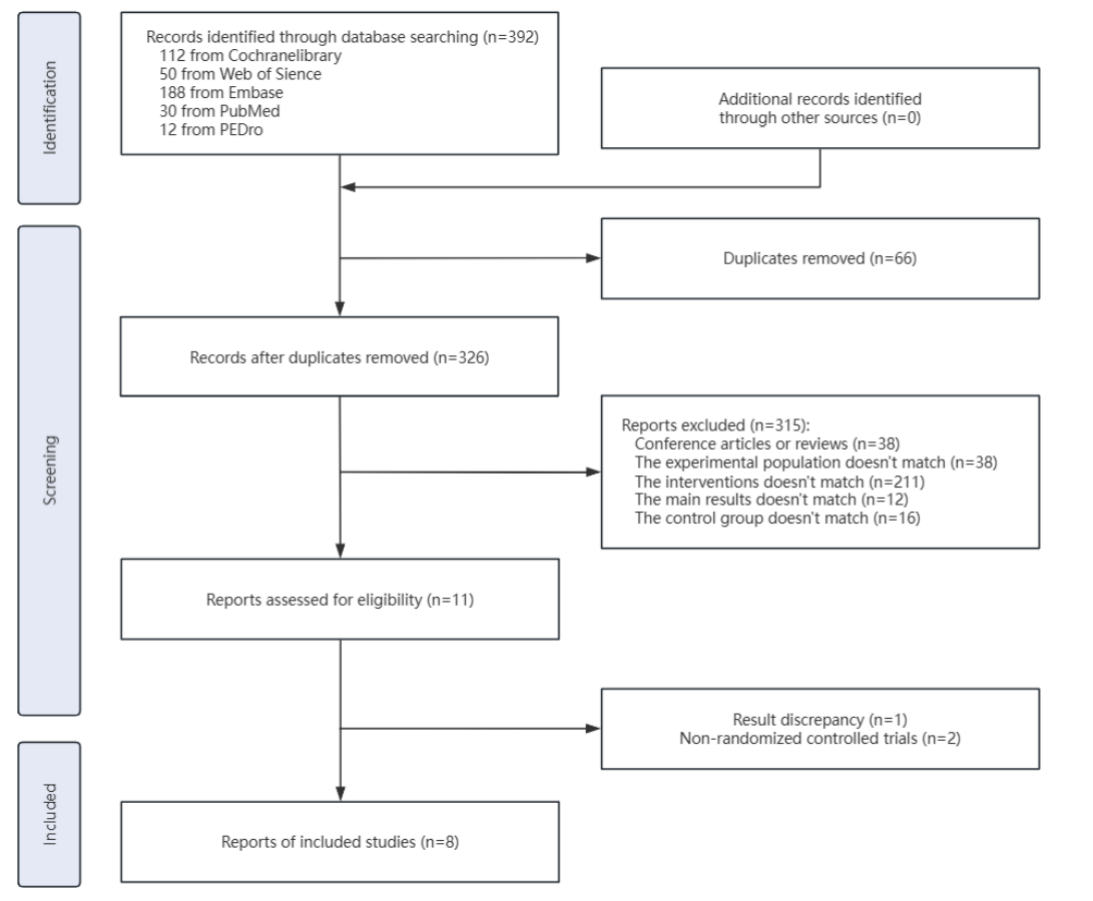
PRISMA flow chart of study selection.

Figure 1 describes the workflow in selecting the studies suitable for the intended analysis. The database search with the specified query returned 370 articles in total, in which 58 were duplicates and removed directly. The titles and abstracts of the remaining items were screened, and 36 conference articles were removed. Further examination identified studies with ineligible subjects (36), non-matching interventions (206), inconsistent primary outcomes (12), and inadequate study designs (11) were eliminated. These 11 studies included non-randomized trials, crossover designs without appropriate washout periods (<4 weeks), and case reports, which did not meet our inclusion criteria for parallel-group RCTs. The 11 remaining articles were perused in full, and among them, two did not carry out randomized controlled trials and one did not have sufficient outcome measures. The final meta-analysis thus included eight randomized controlled trials, which comprised 249 MS patients (Ghannadi et al., 2022; Westerdahl et al., 2015; Smeltzer, Lavietes & Cook, 1996; Silverman et al., 2017; Mutluay et al., 2007; Klefbeck & Hamrah Nedjad, 2003b; Gosselink et al., 2000; Fry et al., 2007). As all data in the systematic review were derived from randomized controlled trials, this work did not directly involve the recruitment of patients or the collection of their information, and there was no need for ethical approval or consent statements.

Table 1 provides a comprehensive summary of the characteristics of the eight reviewed works. All were randomized controlled trials published between 1996 and 2022 and written in English. Three were from the United States, two were from Sweden, and one each was from Iran, Belgium, and Turkey. They all assessed the EDSS scores of the patients at baseline to quantify neurological dysfunction, and four classified the disease subtypes (*i.e.,* relapsing-remitting, secondary progressive, primary progressive, progressive-relapsing). Seven measured MEP (Ghannadi et al., 2022; Westerdahl et al., 2015; Smeltzer, Lavietes & Cook, 1996; Silverman et al., 2017; Mutluay et al., 2007; Klefbeck & Hamrah Nedjad, 2003b; Gosselink et al., 2000; Fry et al., 2007) and MIP (Ghannadi et al., 2022; Westerdahl et al., 2015; Smeltzer, Lavietes & Cook, 1996; Mutluay et al., 2007; Klefbeck & Hamrah Nedjad, 2003b; Gosselink et al., 2000; Fry et al., 2007). Six measured FVC (Ghannadi et al., 2022; Westerdahl et al., 2015; Smeltzer, Lavietes & Cook, 1996; Mutluay et al., 2007; Gosselink et al., 2000; Fry et al., 2007), five measured FEV_1_ (Ghannadi et al., 2022; Westerdahl et al., 2015; Mutluay et al., 2007; Fry et al., 2007), and three assessed FEV_1_/FVC (Ghannadi et al., 2022; Mutluay et al., 2007; Fry et al., 2007).

**Table 1:** Clinical information of the included patients.

**Trial**	**Group size** ^a^	**Age (years)** ^b^ ^,^ ^a^	**EDSS score** ^b^ ^,^ ^a^	**Disease type** ^a^	**Intervention and control protocols**	**Outcome** ^a^
Fry et al., 2007 (USA)	I: 20	50 ± 9.1	3.96 ± 1.80	10 RR, 3 SP, 4 PP, 2 PR^c^	10 weeks of home IMT^a^ Exercise, 15 sets/times, 3 times daily	FVC, MIP, MEP, FEV_1_, FEV_1_/FVC
	C: 21	46.2 ± 9.4	3.36 ± 1.47	16 RR, 4 SP, 1 PP, 1 PR^c^	No intervention	
Klefbeck & Hamrah Nedjad, 2003a and Klefbeck & Hamrah Nedjad, 2003b (Sweden)	I: 7	46 ± 3	7.5 [6.5, 8.0]	N/A	35 days of patient-based 40%–60% PI_max_ training, twice daily (at least 4 h apart), 10 min each time	PI_max_, PE_max_
	C: 8	52.5 ± 5.75	8.0 [6.5, 9.0]	N/A	No intervention	
Ghannadi et al., 2022 (Iran)	I: 19	36.47 ± 7.62	3.52 ± 0.92	all RR	8 weeks of RMT^a^ with a threshold resistance device, twice daily, 3 sets each time, 15 repetitions per set	PI_max_, PE_max_, FVC, FEV_1_, FEV_1_/FVC
	C: 17	39.36 ± 9.83	3.07 ± 0.59	all RR	No intervention	
Westerdahl et al., 2015 (Sweden)	I: 23	55 ± 12	5.0	11 RR, 1 PP, 11 SP	2 months of breathing exercise with a positive expiratory pressure device, twice daily, 30 slow deep breaths each time	FVC, MIP, MEP, FEV_1_
	C: 25	56 ± 9	4.5	9 RR 1 PP, 15 SP	No intervention	
Mutluay et al., 2007 (Turkey)	I: 20	40.3 ± 6	4.85 ± 1.3	4 RR, 5 PP, 11 SP	6 weeks of breathing-enhanced upper extremity exercises at home, once daily, 30 min each time	PI_max_, PE_max_, FVC, FEV_1_, FEV_1_/FVC
	C: 20	38.1 ± 7	4.18 ± 1.7	8 RR, 3 PP, 9 SP	No intervention	
Gosselink et al., 2000 (Belgium)	I: 9	59 ± 14	8 [7, 9]	N/A	3 months of expiratory resistance training, twice daily, 15 contractions each time	FVC, PI_max_, PE_max_
	C: 9	54 ± 13	8.5 [8, 9.5]	N/A	No intervention	
Smeltzer, Lavietes & Cook, 1996 (USA)	I: 10	N/A	[6.5, 9.5]	N/A	6 months of training using an expiratory threshold device, twice daily (at least 4 h apart), 3 sets each time (5 min break between groups), 15 per set	PI_max_, PE_max_
	C: 5	N/A	[6.5, 9.5]	N/A	3 months of sham training using the same device without training load	
Silverman et al., 2017 (USA)	I: 20	N/A	5.48 ± 1.7	N/A	6 weeks of exercise using an experimental pressure threshold trainer, five days each week (150 exercises in total), about 20 min each day, 5 sets of 5 each time	MEP
	C: 16	N/A	5.5 ± 1.5	N/A	Identical training protocol using the same device without an internal pressure threshold spring	

**Notes.**

a I, intervention group; C, control group; EDSS, expanded disability status scale; RR, relapsing-remitting; PP, primary progressive; SP, secondary progressive; PR, progressive relapsing; IMT, inspiratory muscle strength training; RMT, respiratory muscle strength training; MIP (PI_max_), maximal inspiratory pressure; MEP (PE_max_), maximal expiratory pressure; FEV_1_, forced expiratory volume in one second; FVC, forced vital capacity; FEV_1_/FVC, ratio of forced expiratory volume in one second to forced vital capacity.

b Expressed as mean ± standard deviation or mean [minimum, maximum].

c Numbers reproduced from the source article.

### Quality assessment

**Figure 2:**
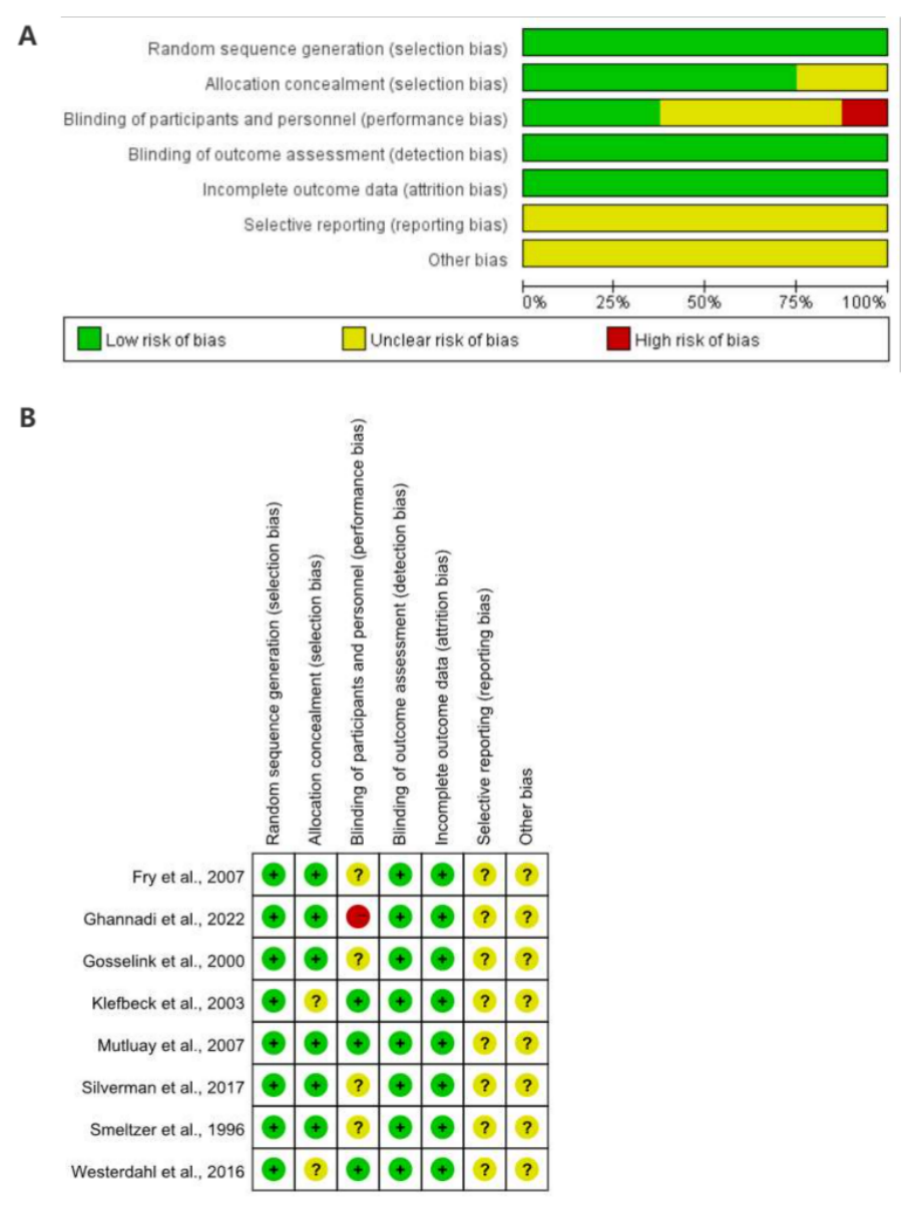
(A) Risk of bias graph. (B) Risk of bias summary.

Figure 2 shows the risk of bias assessment for the included trials. All studies described the randomization process (100%) since only randomized controlled trials were included. Six studies explicitly mentioned implementing allocation concealment (75%), but the other two did not provide sufficient detail. Three studies employed blinding protocols for both patients and trial personnel (37.5%), while one did not, and the other four provided no details. In all studies, the outcome assessors and the data analysts were blinded (100%). The attrition bias (incomplete outcome data) was rated low for five studies (62.5%). Six studies were deemed free of reporting bias (75%), and for the other two, the risk of selective reporting was considered unclear.

### The effect of RMT on respiratory muscle strength

#### The effect of RMT on MEP

**Figure 3:**
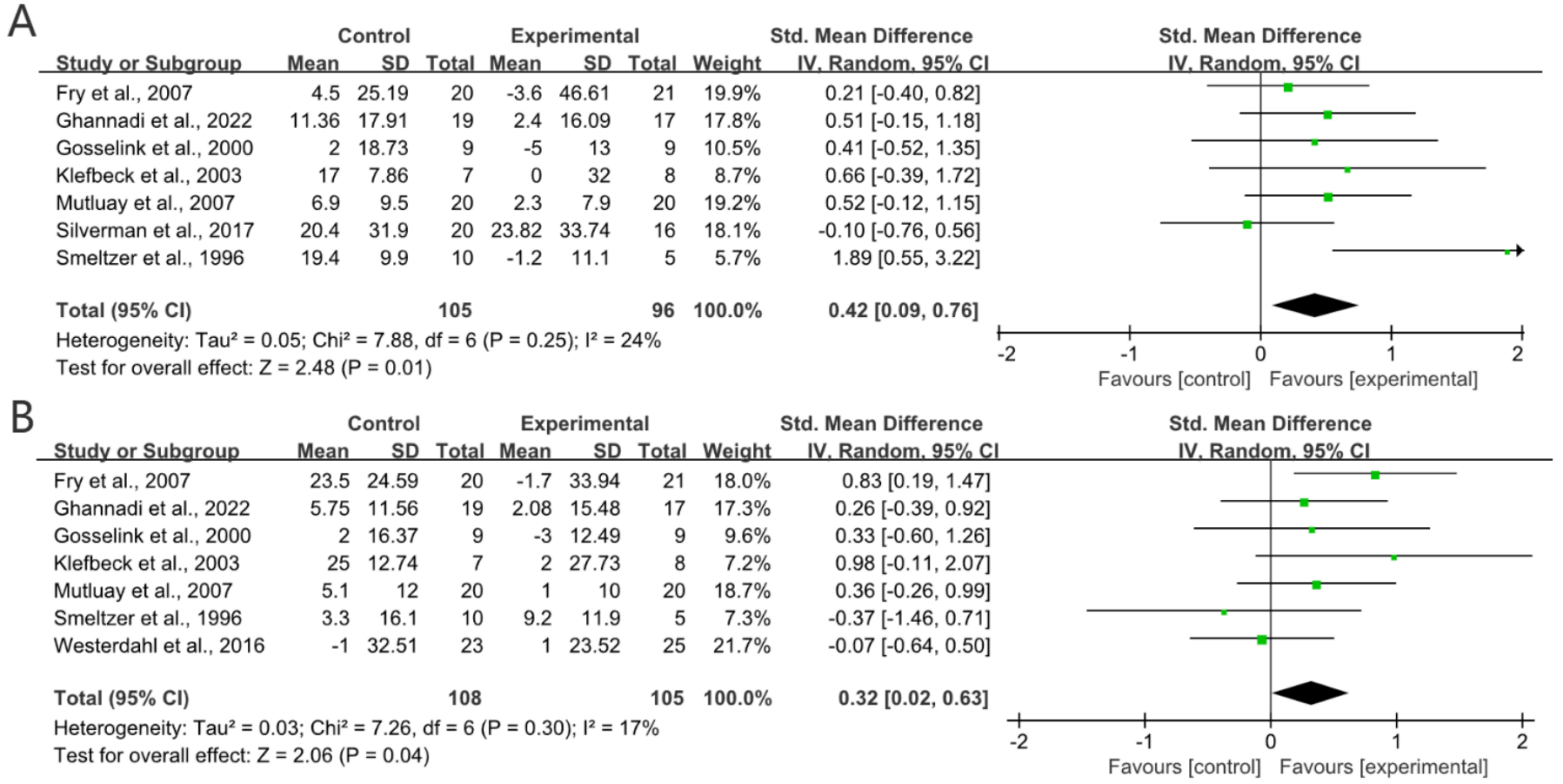
(A) Numerical analysis of patient MEP (B) Numerical analysis of patient MIP.

Seven studies used MEP to assess respiratory muscle strength (Ghannadi et al., 2022; Westerdahl et al., 2015; Smeltzer, Lavietes & Cook, 1996; Silverman et al., 2017; Mutluay et al., 2007; Klefbeck & Hamrah Nedjad, 2003b; Gosselink et al., 2000; Fry et al., 2007). Compared to the controls, MS patients receiving RMT exhibited significantly improved respiratory muscle strength. The standardized mean difference (SMD) was 0.42, and the average increase in MEP was 10%–15% (Fig. 3A). Smeltzer, Lavietes & Cook (1996) found an exceedingly high effect size of 1.89, but Silverman et al. (2017) found a negative effect size of −0.10.

#### The effect of RMT on MIP

Seven trials measured MIP to evaluate the function of the inspiratory muscles (Ghannadi et al., 2022; Westerdahl et al., 2015; Smeltzer, Lavietes & Cook, 1996; Mutluay et al., 2007; Klefbeck & Hamrah Nedjad, 2003b; Gosselink et al., 2000; Fry et al., 2007). Compared to the control, MS patients receiving RMT exhibited significantly improved inspiratory muscle function (Fig. 3B). Heterogeneity testing revealed no significant between-study variance (SMD = 0.32; 95% CI [0.02–0.63]; *P* = 0.04; *I*^2^ = 17%). Klefbeck & Hamrah Nedjad (2003b) found the highest SMD of 0.98, and Fry et al. (2007) had a high SMD of 0.83. In contrast, Smeltzer, Lavietes & Cook (1996) and Westerdahl et al. (2015) found a negative effect size of −0.37 and −0.07, respectively.

### The effect of RMT on pulmonary function

**Figure 4:**
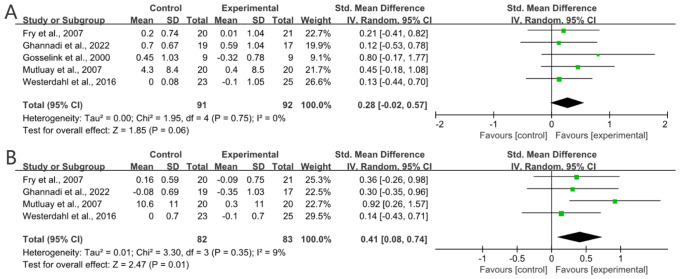
(A) Numerical analyses of patient FVC and (B) Numerical analyses of patient FEV1.

Five studies measured FVC to assess the capacity for ventilatio (Ghannadi et al., 2022; Westerdahl et al., 2015; Mutluay et al., 2007; Gosselink et al., 2000; Fry et al., 2007). Figure 4A shows that RMT did not improve the FVC of MS patients in a statistically significant manner (SMD = 0.28; 95% CI [−0.02–0.57]; *P* = 0.06; *I*^2^ = 0%.

Four studies measured FEV_1_ to assess lung volume and function (Ghannadi et al., 2022; Westerdahl et al., 2015; Mutluay et al., 2007; Fry et al., 2007). Figure 4B shows that RMT effectively improved lung volume and respiratory function in MS patients (SMD = 0.41; 95% CI [0.08–0.74]; *P* = 0.01; *I*^2^ = 9%).

**Figure 5:**
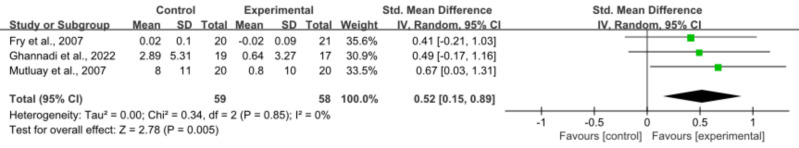
Numerical analysis of patient FVC/FEV1.

Three studies measured FEV_1_/FVC to assess the degree of airflow restriction in the lungs (Ghannadi et al., 2022; Mutluay et al., 2007; Fry et al., 2007). Figure 5 shows that RMT effectively mitigated airflow restriction in the lungs of MS patients (SMD = 0.52; 95% CI [0.15–0.89]; *P* = 0.005; *I*^2^ = 0%).

## Discussion

### The effect of RMT on the respiratory muscle strength

The strength of expiratory and inspiratory muscles can be characterized by MEP and MIP, respectively, and both are critical objective metrics in assessing the respiratory muscle strength in MS patients (Polkey & Man, 2017; De la Maza et al., 2018; Evans & Whitelaw, 2009). Our meta-analysis confirms that RMT significantly improves both MEP (SMD = 0.42) and MIP (SMD = 0.32). This reflects functional adaptation and higher neuromuscular efficiency.

The clinical importance of these findings extends beyond the statistical improvements. Enhanced expiratory strength (MEP) is directly correlated with a more effective cough (Monge-Martínez et al., 2025), which is critical for airway clearance and reducing the risk of aspiration pneumonia—a primary cause of mortality in advanced MS (Jones, Enright & Busse, 2012; Wei, 2014). Furthermore, improved inspiratory muscle function (MIP) may alleviate the subjective sensation of dyspnea (shortness of breath) and has been linked to reductions in overall fatigue (Abasıyanık et al., 2020), a pervasive and debilitating symptom in MS. Therefore, RMT represents a targeted, non-pharmacological intervention that can address life-threatening complications and improve daily quality of life.

Klefbeck & Hamrah Nedjad (2003a) found that in MS patients, EMT resulted in an 18% increase in MEP, and the enhanced control over the active expiratory phase had a statistically significant correlation with the Functional Independence Measure scores. That is, EMT not only improved the respiratory biomechanics but also enhanced the functional autonomy of MS patients.

Current evidence indicates that RMT improves the strength and endurance of the respiratory muscles through both long-term structural modifications of the musculature such as muscle fiber remodeling that underpin endurance, and the rapid enhancement of neuromuscular coordination *via* neural adaptations. Extended RMT triggers the structural remodeling of respiratory muscles, including type II muscle fiber cross-sectional area expansion (*i.e.,* muscular hypertrophy) and mitochondrial biogenesis enhancement. These intrinsic muscular adaptations directly support sustained improvements in respiratory muscle strength and endurance, ultimately improving the respiratory function. Although there are currently no studies in MS patients that focus specifically on how RMT induces muscle hypertrophy, it has been reported in patients with chronic obstructive pulmonary disease that a randomized controlled trial demonstrated an 8-week threshold-loaded IMT protocol significantly increased diaphragm thickness and improved 6-minute walking distance (6MWD) and MIP (Ichiba et al., 2023). Enright et al. (2006) reported that an 8-week IMT protocol increased the diaphragmatic mitochondrial density by 32% in MS patients, and the change was positively correlated with improvements in MIP (*r* = 0.71).

Aside from muscle remodeling, RMT also augments respiratory muscle strength through neural adaptation (Göhl et al., 2016). In MS patients, immune-mediated axonal demyelination compromises neuromuscular transmission efficiency to cause respiratory muscle weakness, which manifests as reduced force generation, hypertonicity, and progressive muscular atrophy (Wei, 2014; Owens, 2016). RMT can mediate respiratory functions through neural adaptation mechanisms such as enhanced motor unit recruitment efficiency, increased firing rates, and improved synchronization of motor units (Seynnes, De Boer & Narici, 2007). These neural adaptation mechanisms (*i.e.,* enhanced neural drive to muscles) need to be considered in evaluating the efficacy of RMT (Matelot et al., 2013). This is particularly relevant for MS patients, in whom impaired neural drive—manifesting as reduced activation of respiratory muscles—is a key component of the disease. Systematic RMT has been shown to reverse the neurodegenerative process (Mahalakshmi et al., 2020; Doust et al., 2014). It activates respiratory muscle groups by applying a threshold load, which requires the patient to generate a specific, preset pressure to initiate airflow. This process enhances the central neural drive, and improves motor unit synchronization, thereby optimizing neuromuscular coordination and facilitating the functional adaptation of the respiratory system. Gosselink et al. (2000) demonstrated that an 8-week IMT protocol significantly improved the synchronization of diaphragmatic electromyographic (EMG) activity in MS patients and found a 22% average increase in MIP, indicating that neural adaptations play a critical role in early-phase strength gains (Smeltzer, Lavietes & Cook, 1996). The heterogeneity in the training protocol across studies (*e.g.*, load intensity, duration) may influence the relative contributions of neural *versus* structural adaptations.

Among the eight reviewed trials, Smeltzer, Lavietes & Cook (1996) reported the highest effect size for MEP (SMD = 1.89), although the wide confidence interval (0.55–3.22) raises some concerns about the accuracy of the effect size estimation. This finding can be explained by the principle of task specificity: the expiratory threshold device provided a targeted resistive load to the expiratory muscles, leading to a specific improvement in expiratory strength. Consequently, the small absolute improvement observed in these patients with advanced disease and poor baseline function may represent a substantial relative gain. Conversely, the same study reported the lowest effect size for MIP (SMD = −0.37), which is consistent with task specificity, as an expiratory-specific intervention would not be expected to markedly improve inspiratory strength. The wide 95% CI for MEP suggests high uncertainty, likely due to the small sample size.

In contrast, Silverman et al. (2017) reported a negative effect size for MEP (SMD = −0.10). We hypothesize that this lack of efficacy may be attributed to their discontinuous training protocol (five days per week), which may have provided an insufficient stimulus frequency for robust neural and muscular adaptations.

Klefbeck & Hamrah Nedjad (2003b) reported the highest effect size observed for MIP (SMD = 0.98), but overestimation cannot be excluded in view of the wide confidence interval (−0.11 to 2.07). In addition, their sample size is small, with only seven participants in the intervention group and eight in the control group. The high SMD may be attributed to the inclusion of severely ill patients, with a mean EDSS score of 7.5, as the lower baseline provides greater room for improvement. In contrast, the high SMD (0.83, 95% CI [0.19–1.47]) for MIP reported by Fry et al. (2007) appears more convincing, as their study included more patients (41 in total) that have a mild disease (mean EDSS score = 3.96). Their patients do not suffer from significant respiratory muscular atrophy, and the preserved muscle integrity may have facilitated respiratory muscle adaptations. Westerdahl et al. (2015) found a slight negative effect size on MIP (SMD = −0.07) that does not necessarily disprove the benefits of RMT, since only two of the 23 people in the intervention group had a lower MIP value than the control. It is possible that these two people generate extreme values, and if those two individuals were removed, the SMD for MIP might have been positive.

The reviewed trials generally validate the benefits of RMT, although their results are not uniform. It must be noted that RMT settings such as training frequency, intensity, and duration all influence the effectiveness of the treatment. The intensity and frequency of RMT can impact the strength and endurance of respiratory muscles. By improving the efficiency of ventilation, RMT can thereby enhance, gas exchange, enhancing blood oxygen saturation, and alleviating patient discomfort (Silva et al., 2019; Sapienza, 2008; Ramli et al., 2023). To ensure individualized and optimized treatment regimens, a thorough assessment of the patient’s clinical status and the pathophysiological severity of the disease is needed before setting the training parameters. It is crucial to manage training parameters carefully, as excessive, poorly monitored training carries a potential risk of inducing significant muscle fatigue or weakness, which could be detrimental in this population (Ghannadi et al., 2022; Fry et al., 2007; Del Corral et al., 2023).

### The effect of RMT on pulmonary function

Regarding pulmonary function, our analysis found that RMT significantly improved FEV1 (SMD = 0.41) and FEV1/FVC (SMD = 0.52), but not FVC (SMD = 0.28, *P* = 0.06). Forced vital capacity (FVC) is the total amount of air a person can forcibly exhale after taking the deepest breath possible. It is useful for detecting restrictive lung diseases such as pulmonary fibrosis where lung expansion is limited. Forced expiratory volume in one second (FEV_1_) assesses the volume of air exhaled in the first second of the FVC maneuver and is an important indicator of airflow obstruction. The FEV_1_/FVC ratio helps differentiate between obstructive and restrictive lung diseases (Hankinson et al., 2015). A reduced FEV_1_/FVC is characteristic of obstructive diseases, while a normal or increased FEV_1_/FVC is often seen in restrictive diseases because both FEV_1_ and FVC are proportionally reduced (Hankinson et al., 2015; Nathan et al., 2021; Rivero-Yeverino, 2019; Almaasfeh et al., 2023). These metrics together provide valuable insights into lung function and aid in diagnosing various pulmonary conditions.

The increase in FEV_1_ due to RMT (SMD = 0.41, 95% CI [0.08–0.74], *P* = 0.01) can be readily related to enhanced respiratory muscle strength. By increasing the strength and neural adaptation of the expiratory muscles, RMT increases MEP, thus directly improving the flow in the initial expiratory phase (Brennan et al., 2022; Willis, 2023; Burge, 1992; Aguirre et al., 2023).

Although the five studies that assessed FVC in aggregate suggested that RMT does not have a significant impact on FVC (SMD = 0.28, *P* = 0.06), this lack of significance may be explained by the principle of training specificity. FVC is a ballistic, forceful expiratory maneuver. However, several of the included trials did not specifically train for this. For example, Westerdahl et al. (2015) and Smeltzer, Lavietes & Cook (1996) used slow deep breathing with PEP, and Fry et al. (2007) and Polkey & Man (2017) used inspiratory training (IMT). It is therefore not surprising that interventions lacking a strong, ballistic expiratory component failed to improve FVC, even if they improved other measures like MIP. This highlights the importance of matching the RMT modality to the desired functional outcome.

Among mild MS patients, the low confidence in FVC measurements may stem from the challenges in standardizing testing maneuvers, as individuals with lower disability levels (*e.g.*, EDSS ≤ 5.0) often retain greater physical capacity and the expiratory efforts during testing can be inconsistent. Due to their preserved motor function, mild MS patients have variable exertion patterns ranging from overexertion to postural adjustments, which may complicate the accurate measurement of FVC. In contrast, severe MS patients (EDSS > 5.0) are typically wheelchair-bound or bedridden, and because of the limited mobility, they maintain fixed postures during testing. As a result, their expiratory efforts are much more standardized and there is less error. Katz et al. (2018) conducted a systematic analysis on the relationship between patient positioning and pulmonary function, revealing that the lateral decubitus position was associated with a decline in FVC. In contrast, the supine position increased elastic load on the lungs, though FVC might improve due to enhanced diaphragmatic activity. The exclusion of mild cases can decrease baseline heterogeneity and amplify detectable treatment effects, as severe patients exhibit more pronounced respiratory muscle dysfunction. When the mild MS patients (EDSS score ≤ 5.0) were excluded from the analysis, the SMD of FVC increased from 0.28 to 0.36, although the 95% CI still included zero (Ghannadi et al., 2022; Westerdahl et al., 2015). Presumably, the improvement of FVC requires prolonged training duration or individualized intensity adjustment.

The increase in FEV_1_/FVC due to RMT (SMD = 0.52, 95% CI [0.15–0.89], *P* = 0.005) cannot be interpreted as the alleviation of airway obstruction, as it results from the improvement in FEV_1_ alone without any variation in FVC. In other words, RMT primarily targets the functions of respiratory muscle rather than the structural modifications of the airway (Enright et al., 2006). Pulmonary function metrics need to be interpreted comprehensively and not in isolation.

## Limitations

First, some trials did not measure subjective outcome measures such as fatigue, endurance, psychological status, and quality of life, even though those factors are very important for understanding the overall impact of RMT on patients. For those that did, the methodology varied. For example, fatigue was assessed by Ghannadi et al. (2022) using the Modified Fatigue Impact Scale (MFIS) but by Klefbeck & Hamrah Nedjad (2003a) and Klefbeck & Hamrah Nedjad (2003b) using the Fatigue Severity Scale (FSS). Therefore, in this work, we focused only on objective outcome measures that were adopted in the reviewed trials most commonly.

Second, and perhaps most significantly, our analysis did not stratify results by RMT type (*i.e.,* inspiratory, expiratory, or combined training) or by baseline disease severity (EDSS). These are major potential confounders. For instance, an intervention’s effect on MEP would likely be greater with EMT than IMT. Unfortunately, the small number of included studies, combined with the varied reporting of these parameters, made formal subgroup analyses or meta-regression statistically unfeasible. This limits our ability to draw conclusions about which RMT protocol is most effective for which patient subgroup.

Third, there is currently no standardized guideline for RMT, and the RMT protocol varied across trials. The reviewed studies adopted different parameters such as frequency, intensity, duration, *etc.*, and these variations may have contributed to the changes in treatment outcomes.

Nevertheless, the current body of evidence remains in a developmental stage. To further consolidate these findings and provide more robust guidance for clinical practice, future well-designed, large-scale randomized controlled trials are warranted. These studies must prioritize establishing standardized RMT protocols, stratifying analyses by MS subtype and EDSS score, and directly comparing the efficacy of IMT, EMT, and combined RMT. Furthermore, future trials should include long-term follow-up and incorporate crucial subjective outcomes, such as fatigue (*e.g.*, MFIS), sleep quality, and health-related quality of life, to fully capture the clinical impact of RMT.

## Conclusions

This systematic review demonstrates that respiratory muscle training (RMT) is a beneficial intervention for enhancing respiratory muscle strength, particularly maximal expiratory and inspiratory pressures (MEP and MIP), and improving ventilatory function (FEV1, FEV1/FVC) in patients with multiple sclerosis (MS). These improvements have important clinical implications, including the potential to reduce pneumonia risk and alleviate symptoms of dyspnea and fatigue.

It is therefore recommended that RMT be incorporated as a cornerstone of adjunctive therapy within comprehensive rehabilitation plans for MS patients. While the current evidence base is characterized by methodological heterogeneity and a need for more long-term data, the consistent positive findings regarding core respiratory outcomes provide a compelling rationale for its broader clinical application. To solidify this foundation, future research must prioritize the execution of large-scale, rigorous randomized controlled trials aimed at establishing standardized protocols and elucidating the long-term impact of RMT across diverse patient populations.

##  Supplemental Information

10.7717/peerj.20876/supp-1 Supplemental Information 1 PRISMA 2020 checklist

10.7717/peerj.20876/supp-2 Supplemental Information 2 Audience of the article

10.7717/peerj.20876/supp-3 Supplemental Information 3 Search history

10.7717/peerj.20876/supp-4 Supplemental Information 4 Meta-Analysis Rationale

## Additional Information and Declarations

### Data Availability

The following information was supplied regarding data availability: This is a systematic review/meta-analysis.
