# Supplementary material for: Improving respiratory muscle strength in patients with multiple sclerosis through respiratory muscle training: a systematic review and meta-analysis
Source: PeerJ. 2026 Apr 9;14:e20876. doi: 10.7717/peerj.20876 (PMC13070315; doi:10.7717/peerj.20876)
Supplement: Supplemental Information 2 [file peerj-14-20876-s002.docx]

The primary audience for this article includes medical researchers, clinicians, and physiotherapists, particularly professionals specializing in the rehabilitation of patients with multiple sclerosis (MS). Through its systematic review and meta-analysis, the study provides evidence-based support for evaluating the effectiveness of respiratory muscle training (RMT) in improving respiratory muscle strength and pulmonary function in MS patients, offering valuable insights for designing intervention programs in clinical practice. While the article focuses on academic research, its conclusions hold potential relevance for MS patients and their families interested in non-pharmacological interventions to enhance quality of life.
