# Supplementary material for: Improving respiratory muscle strength in patients with multiple sclerosis through respiratory muscle training: a systematic review and meta-analysis
Source: PeerJ. 2026 Apr 9;14:e20876. doi: 10.7717/peerj.20876 (PMC13070315; doi:10.7717/peerj.20876)
Supplement: Supplemental Information 3 [file peerj-14-20876-s003.pdf]

Entitlements	#	Search Query	Database	Results	Date Run
- WOS.IC: 1993 to 2024- WOS.CCR: 1985 to 2024- WOS.SCI: 2013 to 2024	1	ALL=(Multiple Sclerosis)	Web of Science Core Collection	94365	Mon Mar 18 2024 21:59:05 GMT+0800 (中国标准时间)
- WOS.IC: 1993 to 2024- WOS.CCR: 1985 to 2024- WOS.SCI: 2013 to 2024	2	ALL=(Sclerosis, Multiple OR Sclerosis, Disseminated OR Disseminated Sclerosis OR MS (Multiple Sclerosis) OR Multiple Sclerosis, Acute Fulminating)	Web of Science Core Collection	94446	Mon Mar 18 2024 21:59:29 GMT+0800 (中国标准时间)
- WOS.IC: 1993 to 2024- WOS.CCR: 1985 to 2024- WOS.SCI: 2013 to 2024	3	(ALL=(Sclerosis, Multiple OR Sclerosis, Disseminated OR Disseminated Sclerosis OR MS (Multiple Sclerosis) OR Multiple Sclerosis, Acute Fulminating)) AND ALL=(Multiple Sclerosis)	Web of Science Core Collection	94365	Mon Mar 18 2024 21:59:43 GMT+0800 (中国标准时间)
- WOS.IC: 1993 to 2024- WOS.CCR: 1985 to 2024- WOS.SCI: 2013 to 2024	4	ALL=(Breathing Exercises)	Web of Science Core Collection	4476	Mon Mar 18 2024 22:00:07 GMT+0800 (中国标准时间)
- WOS.IC: 1993 to 2024- WOS.CCR: 1985 to 2024- WOS.SCI: 2013 to 2024	5	ALL=(Exercise, Breathing OR Respiratory Muscle Training OR Muscle Training, Respiratory OR Training, Respiratory Muscle)	Web of Science Core Collection	7576	Mon Mar 18 2024 22:00:30 GMT+0800 (中国标准时间)
- WOS.IC: 1993 to 2024- WOS.CCR: 1985 to 2024- WOS.SCI: 2013 to 2024	6	(ALL=(Exercise, Breathing OR Respiratory Muscle Training OR Muscle Training, Respiratory OR Training, Respiratory Muscle)) AND ALL=(Breathing Exercises)	Web of Science Core Collection	4470	Mon Mar 18 2024 22:00:43 GMT+0800 (中国标准时间)
- WOS.IC: 1993 to 2024- WOS.CCR: 1985 to 2024- WOS.SCI: 2013 to 2024	7	(((ALL=(Exercise, Breathing OR Respiratory Muscle Training OR Muscle Training, Respiratory OR Training, Respiratory Muscle)) AND ALL=(Breathing Exercises)) AND ALL=(Sclerosis, Multiple OR Sclerosis, Disseminated OR Disseminated Sclerosis OR MS (Multiple S[illegible]	Web of Science Core Collection	41	Mon Mar 18 2024 22:01:32 GMT+0800 (中国标准时间)

Search number	Query	Sort By	Filters	Search Details	Results	Time
8	((Multiple Sclerosis) OR (Sclerosis, Multiple OR Sclerosis, Disseminated OR Disseminated Sclerosis OR MS (Multiple Sclerosis) OR Multiple Sclerosis, Acute Fulminating)) AND ((Breathing Exercises) OR (Exercise, Breathing OR Respiratory Muscle Training OR Muscle Training, Respiratory OR Training, Respiratory Muscle))		Randomized Controlled Trial	(("multiple sclerosis"[MeSH Terms] OR ("multiple"[All Fields] AND "sclerosis"[All Fields]) OR "multiple sclerosis"[All Fields] OR ((("multiple sclerosis"[MeSH Terms] OR ("multiple"[All Fields] AND "sclerosis"[All Fields]) OR "multiple sclerosis"[All Fields] OR ("sclerosis"[All Fields] AND "multiple"[All Fields]) OR "sclerosis multiple"[All Fields] OR ("multiple sclerosis"[MeSH Terms] OR ("multiple"[All Fields] AND "sclerosis"[All Fields]) OR "multiple sclerosis"[All Fields] OR ("sclerosis"[All Fields] AND "disseminated"[All Fields]) OR "sclerosis disseminated"[All Fields] OR ("multiple sclerosis"[MeSH Terms] OR ("multiple"[All Fields] AND "sclerosis"[All Fields]) OR "multiple sclerosis"[All Fields] OR ("disseminated"[All Fields] AND "sclerosis"[All Fields]) OR "disseminated sclerosis"[All Fields]) OR ("ms"[Journal] OR "ms"[Journal] OR "med sci paris"[Journal] OR "ms"[All Fields])) AND ("multiple sclerosis"[MeSH Terms] OR ("multiple"[All Fields] AND "sclerosis"[All Fields]) OR "multiple sclerosis"[All Fields])) OR ("multiple sclerosis"[MeSH Terms] OR ("multiple"[All Fields] AND "sclerosis"[All Fields]) OR "multiple sclerosis"[All Fields] OR ("multiple"[All Fields] AND "sclerosis"[All Fields] AND "acute"[All Fields] AND "fulminating"[All Fields]) OR "multiple sclerosis acute fulminating"[All Fields])))) AND ("breathing exercises"[MeSH Terms] OR ("breathing"[All Fields] AND "exercises"[All Fields]) OR "breathing exercises"[All Fields] OR ("breathing exercises"[MeSH Terms] OR ("breathing"[All Fields] AND "exercises"[All Fields]) OR "breathing exercises"[All Fields] OR ("exercise"[All Fields] AND "breathing"[All Fields]) OR "exercise breathing"[All Fields] OR ("breathing exercises"[MeSH Terms] OR ("breathing"[All Fields] AND "exercises"[All Fields]) OR "breathing exercises"[All Fields] OR ("respiratory"[All Fields] AND "muscle"[All Fields] AND "training"[All Fields]) OR "respiratory muscle training"[All Fields]) OR ("breathing exercises"[MeSH Terms] OR ("breathing"[All Fields] AND "exercises"[All Fields]) OR "breathing exercises"[All Fields] OR ("muscle"[All Fields] AND "training"[All Fields] AND "respiratory"[All Fields]) OR "muscle training respiratory"[All Fields]) OR ("breathing exercises"[MeSH Terms] OR ("breathing"[All Fields] AND "exercises"[All Fields]) OR "breathing exercises"[All Fields] OR ("training"[All Fields] AND "respiratory"[All Fields] AND "muscle"[All Fields]) OR "training respiratory muscle"[All Fields]))))) AND (randomizedcontrolledtrial[Filter])	19	8:32:17
7	((Multiple Sclerosis) OR (Sclerosis, Multiple OR Sclerosis, Disseminated OR Disseminated Sclerosis OR MS (Multiple Sclerosis) OR Multiple Sclerosis, Acute Fulminating)) AND ((Breathing Exercises) OR (Exercise, Breathing OR Respiratory Muscle Training OR Muscle Training, Respiratory OR Training, Respiratory Muscle))			("multiple sclerosis"[MeSH Terms] OR ("multiple"[All Fields] AND "sclerosis"[All Fields]) OR "multiple sclerosis"[All Fields] OR ((("multiple sclerosis"[MeSH Terms] OR ("multiple"[All Fields] AND "sclerosis"[All Fields]) OR "multiple sclerosis"[All Fields] OR ("sclerosis"[All Fields] AND "multiple"[All Fields]) OR "sclerosis multiple"[All Fields] OR ("multiple sclerosis"[MeSH Terms] OR ("multiple"[All Fields] AND "sclerosis"[All Fields]) OR "multiple sclerosis"[All Fields] OR ("sclerosis"[All Fields] AND "disseminated"[All Fields]) OR "sclerosis disseminated"[All Fields] OR ("multiple sclerosis"[MeSH Terms] OR ("multiple"[All Fields] AND "sclerosis"[All Fields]) OR "multiple sclerosis"[All Fields] OR ("disseminated"[All Fields] AND "sclerosis"[All Fields]) OR "disseminated sclerosis"[All Fields]) OR ("ms"[Journal] OR "ms"[Journal] OR "med sci paris"[Journal] OR "ms"[All Fields])) AND ("multiple sclerosis"[MeSH Terms] OR ("multiple"[All Fields] AND "sclerosis"[All Fields]) OR "multiple sclerosis"[All Fields])) OR ("multiple sclerosis"[MeSH Terms] OR ("multiple"[All Fields] AND "sclerosis"[All Fields]) OR "multiple sclerosis"[All Fields] OR ("multiple"[All Fields] AND "sclerosis"[All Fields] AND "acute"[All Fields] AND "fulminating"[All Fields]) OR "multiple sclerosis acute fulminating"[All Fields])))) AND ("breathing exercises"[MeSH Terms] OR ("breathing"[All Fields] AND "exercises"[All Fields]) OR "breathing exercises"[All Fields] OR ("breathing exercises"[MeSH Terms] OR ("breathing"[All Fields] AND "exercises"[All Fields]) OR "breathing exercises"[All Fields] OR ("exercise"[All Fields] AND "breathing"[All Fields]) OR "exercise breathing"[All Fields] OR ("breathing exercises"[MeSH Terms] OR ("breathing"[All Fields] AND "exercises"[All Fields]) OR "breathing exercises"[All Fields] OR ("respiratory"[All Fields] AND "muscle"[All Fields] AND "training"[All Fields]) OR "respiratory muscle training"[All Fields]) OR ("breathing exercises"[MeSH Terms] OR ("breathing"[All Fields] AND "exercises"[All Fields]) OR "breathing exercises"[All Fields] OR ("muscle"[All Fields] AND "training"[All Fields] AND "respiratory"[All Fields]) OR "muscle training respiratory"[All Fields]) OR ("breathing exercises"[MeSH Terms] OR ("breathing"[All Fields] AND "exercises"[All Fields]) OR "breathing exercises"[All Fields] OR ("training"[All Fields] AND "respiratory"[All Fields] AND "muscle"[All Fields]) OR "training respiratory muscle"[All Fields]))))	84	8:32:10

6	(Breathing Exercises) OR (Exercise, Breathing OR Respiratory Muscle Training OR Muscle Tra	"breathing exercises"[MeSH Terms] OR ("breathing"[All Fields] AND "exercises"[All Fields]) OR "breathing exercises"[All Fields] OR ("breathing exercises"[MeSH Terms] OR ("breathing"[All Fields] AND "exercises"[All Fields]) OR "breathing exercises"[All Fields] OR ("exercise"[All Fields] AND "breathing"[All Fields]) OR "exercise breathing"[All Fields] OR ("breathing exercises"[MeSH Terms] OR ("breathing"[All Fields] AND "exercises"[All Fields]) OR "breathing exercises"[All Fields] OR ("respiratory"[All Fields] AND "muscle"[All Fields] AND "training"[All Fields]) OR "respiratory muscle training"[All Fields]) OR ("breathing exercises"[MeSH Terms] OR ("breathing"[All Fields] AND "exercises"[All Fields]) OR "breathing exercises"[All Fields] OR ("muscle"[All Fields] AND "training"[All Fields] AND "respiratory"[All Fields]) OR "muscle training respiratory"[All Fields]) OR ("breathing exercises"[MeSH Terms] OR ("breathing"[All Fields] AND "exercises"[All Fields]) OR "breathing exercises"[All Fields] OR ("training"[All Fields] AND "respiratory"[All Fields] AND "muscle"[All Fields]) OR "training respiratory muscle"[All Fields]))	14,432	8:22:55
5	Exercise, Breathing OR Respiratory Muscle Training OR Muscle Training, Respiratory OR Training, Respiratory Muscle	"breathing exercises"[MeSH Terms] OR ("breathing"[All Fields] AND "exercises"[All Fields]) OR "breathing exercises"[All Fields] OR ("exercise"[All Fields] AND "breathing"[All Fields]) OR "exercise breathing"[All Fields] OR ("breathing exercises"[MeSH Terms] OR ("breathing"[All Fields] AND "exercises"[All Fields]) OR "breathing exercises"[All Fields] OR ("respiratory"[All Fields] AND "muscle"[All Fields] AND "training"[All Fields]) OR "respiratory muscle training"[All Fields]) OR ("breathing exercises"[MeSH Terms] OR ("breathing"[All Fields] AND "exercises"[All Fields]) OR "breathing exercises"[All Fields] OR ("muscle"[All Fields] AND "training"[All Fields] AND "respiratory"[All Fields]) OR "muscle training respiratory"[All Fields]) OR ("breathing exercises"[MeSH Terms] OR ("breathing"[All Fields] AND "exercises"[All Fields]) OR "breathing exercises"[All Fields] OR ("training"[All Fields] AND "respiratory"[All Fields] AND "muscle"[All Fields]) OR "training respiratory muscle"[All Fields])	14,432	8:22:42
4	Breathing Exercises	"breathing exercises"[MeSH Terms] OR ("breathing"[All Fields] AND "exercises"[All Fields]) OR "breathing exercises"[All Fields]	5,798	8:22:22
3	(Multiple Sclerosis) OR (Sclerosis, Multiple OR Sclerosis, Disseminated OR Disseminated Sclerosis OR MS (Multiple Sclerosis) OR Multiple Sclerosis, Acute Fulminating)	"multiple sclerosis"[MeSH Terms] OR ("multiple"[All Fields] AND "sclerosis"[All Fields]) OR "multiple sclerosis"[All Fields] OR ((("multiple sclerosis"[MeSH Terms] OR ("multiple"[All Fields] AND "sclerosis"[All Fields]) OR "multiple sclerosis"[All Fields] OR ("sclerosis"[All Fields] AND "multiple"[All Fields]) OR "sclerosis multiple"[All Fields] OR ("multiple sclerosis"[MeSH Terms] OR ("multiple"[All Fields] AND "sclerosis"[All Fields]) OR "multiple sclerosis"[All Fields] OR ("sclerosis"[All Fields] AND "disseminated"[All Fields]) OR "sclerosis disseminated"[All Fields]) OR ("multiple sclerosis"[MeSH Terms] OR ("multiple"[All Fields] AND "sclerosis"[All Fields]) OR "multiple sclerosis"[All Fields] OR ("disseminated"[All Fields] AND "sclerosis"[All Fields]) OR "disseminated sclerosis"[All Fields]) OR ("ms"[Journal] OR "ms"[Journal] OR "med sci paris"[Journal] OR "ms"[All Fields])) AND ("multiple sclerosis"[MeSH Terms] OR ("multiple"[All Fields] AND "sclerosis"[All Fields]) OR "multiple sclerosis"[All Fields])) OR ("multiple sclerosis"[MeSH Terms] OR ("multiple"[All Fields] AND "sclerosis"[All Fields]) OR "multiple sclerosis"[All Fields] OR ("multiple"[All Fields] AND "sclerosis"[All Fields] AND "acute"[All Fields] AND "fulminating"[All Fields]) OR "multiple sclerosis acute fulminating"[All Fields]))	112,203	8:19:28
2	Sclerosis, Multiple OR Sclerosis, Disseminated OR Disseminated Sclerosis OR MS (Multiple S	"multiple sclerosis"[MeSH Terms] OR ("multiple"[All Fields] AND "sclerosis"[All Fields]) OR "multiple sclerosis"[All Fields] OR ("sclerosis"[All Fields] AND "multiple"[All Fields]) OR "sclerosis multiple"[All Fields] OR ("multiple sclerosis"[MeSH Terms] OR ("multiple"[All Fields] AND "sclerosis"[All Fields]) OR "multiple sclerosis"[All Fields] OR ("sclerosis"[All Fields] AND "disseminated"[All Fields]) OR "sclerosis disseminated"[All Fields]) OR ("multiple sclerosis"[MeSH Terms] OR ("multiple"[All Fields] AND "sclerosis"[All Fields]) OR "multiple sclerosis"[All Fields] OR ("disseminated"[All Fields] AND "sclerosis"[All Fields]) OR "disseminated sclerosis"[All Fields]) OR ("multiple sclerosis"[MeSH Terms] OR ("multiple"[All Fields] AND "sclerosis"[All Fields]) OR "multiple sclerosis"[All Fields] OR ("ms"[All Fields] AND "multiple"[All Fields] AND "sclerosis"[All Fields]) OR "ms multiple sclerosis"[All Fields]) OR ("multiple sclerosis"[MeSH Terms] OR ("multiple"[All Fields] AND "sclerosis"[All Fields]) OR "multiple sclerosis"[All Fields] OR ("multiple"[All Fields] AND "sclerosis"[All Fields] AND "acute"[All Fields] AND "fulminating"[All Fields]) OR "multiple sclerosis acute fulminating"[All Fields])	112,460	8:19:10
1	Multiple Sclerosis	"multiple sclerosis"[MeSH Terms] OR ("multiple"[All Fields] AND "sclerosis"[All Fields]) OR "multiple sclerosis"[All Fields]	112,203	8:18:58

Search Name:
Date Run: 20/03/2024 15:01:21
Comment:
ID Search Hits
#1 MeSH descriptor: [Multiple Sclerosis] explode all trees 5178
#2 MeSH descriptor: [Breathing Exercises] explode all trees 1336
#3 ((Exercise, Breathing) OR (Respiratory Muscle Training) OR (Muscle Training, Respiratory) OR (Training, Respiratory Muscle)):ti,ab,kw (Word variations have been searched) 10566
#4 ((Sclerosis, Multiple) OR (Sclerosis, Disseminated) OR (Disseminated Sclerosis) OR (MS (Multiple Sclerosis)) OR (Multiple Sclerosis, Acute Fulminating)):ti,ab,kw (Word variations have been searched) 12930
#5 #1 or #4 12930
#6 #2 or #3 10696
#7 #5 and #6 114

Embase

Embase session results (22 Mar 2024)

No.	Query	Results
#10	#6 AND #8 AND [randomized controlled trial]/lim	188
#9	#6 AND #8	892
#8	#1 OR #7	189301
#7	'sclerosis, multiple' OR 'sclerosis, disseminated' OR 'disseminated sclerosis' OR 'multiple sclerosis, acute fulminating'	1078
#6	#4 OR #5	97846
#5	'muscle training' OR 'muscle training, respiratory' OR 'training, respiratory muscle'	97497
#4	'respiratory training'	537
#3	#1 OR #2	816212
#2	'sclerosis, multiple' OR 'sclerosis, disseminated' OR 'disseminated sclerosis' OR 'ms' OR 'multiple sclerosis, acute fulminating'	726631
#1	'multiple sclerosis'	189188

Copyright © 2024 Elsevier Limited except certain content provided by third parties.
Embase is a trade mark of Elsevier Life Sciences IP Limited.

Embase | Search | Emtree | Journals | Results | My tools | Sign in

#6 AND #8 AND [2024-2026]/py

Search > | Mapping | Date | Sources | Fields | Quick limits | EBM | Pub. types | Languages | Gender | Age | Animal | Search tips

Results Filters: Sources, Drugs, Diseases, Devices, Floating Subheadings, Age, Gender, Study types, Publication types, Journal titles

History | Save | Delete | Print view | Export | Email | Combine > using And / Or | Collapse

#10	#6 AND #8 AND [2024-2026]/py	0
#9	#6 AND #8	7
#8	#1 OR #7	212,280
#7	'sclerosis, multiple' OR 'sclerosis, disseminated' OR 'disseminated sclerosis' OR 'multiple sclerosis, acute fulminating'	1,167
#6	#4 AND #5	158
#5	'muscle training' OR 'muscle training, respiratory' OR 'training, respiratory muscle'	25,949
#4	'respiratory training'	766
#3	#1 AND #2	113,451
#2	'sclerosis, multiple' OR 'sclerosis, disseminated' OR 'disseminated sclerosis' OR 'ms' OR 'multiple sclerosis, acute fulminating'	821,226
#1	'multiple sclerosis'/exp OR 'multiple sclerosis'	212,165

0 results for search #10 | Set email alert | Search details | Set RSS feed | Index miner

View | Export | Email | Add to Temporary list

Search n	Query	Sort By	Filters	Search Details	Results	Time	Date
6	(((Exercise, Breathing OR Respiratory Muscle Training OR Muscle Training, Respiratory OR Training, Respiratory Muscle) AND (Breathing Exercises)) AND (Sclerosis, Multiple OR Sclerosis, Disseminated OR Disseminated Sclerosis OR MS (Multiple Sclerosis) OR Multiple Sclerosis, Acute Fulminating))) AND (Multiple Sclerosis)	Most Recent	from 2024/3/1 - 3000/12/12	(("breathing exercises"[MeSH Terms] OR ("breathing"[All Fields] AND "exercises"[All Fields]) OR "breathing exercises"[All Fields] OR ("exercise"[All Fields] AND "breathing"[All Fields]) OR "exercise breathing"[All Fields] OR ("breathing exercises"[MeSH Terms] OR ("breathing"[All Fields] AND "exercises"[All Fields]) OR "breathing exercises"[All Fields] OR ("respiratory"[All Fields] AND "muscle"[All Fields] AND "training"[All Fields]) OR "respiratory muscle training"[All Fields]) OR ("breathing exercises"[MeSH Terms] OR ("breathing"[All Fields] AND "exercises"[All Fields]) OR "breathing exercises"[All Fields] OR ("muscle"[All Fields] AND "training"[All Fields] AND "respiratory"[All Fields]) OR "muscle training respiratory"[All Fields]) OR ("breathing exercises"[MeSH Terms] OR ("breathing"[All Fields] AND "exercises"[All Fields]) OR "breathing exercises"[All Fields] OR ("training"[All Fields] AND "respiratory"[All Fields] AND "muscle"[All Fields]) OR "training respiratory muscle"[All Fields])) AND ("breathing exercises"[MeSH Terms] OR ("breathing"[All Fields] AND "exercises"[All Fields]) OR "breathing exercises"[All Fields]) AND ((("multiple sclerosis"[MeSH Terms] OR ("multiple"[All Fields] AND "sclerosis"[All Fields]) OR "multiple sclerosis"[All Fields] OR ("sclerosis"[All Fields] AND "multiple"[All Fields]) OR "sclerosis, multiple"[All Fields] OR ("multiple sclerosis"[MeSH Terms] OR ("multiple"[All Fields] AND "sclerosis"[All Fields]) OR "multiple sclerosis"[All Fields] OR ("sclerosis"[All Fields] AND "disseminated"[All Fields]) OR "sclerosis disseminated"[All Fields]) OR ("multiple sclerosis"[MeSH Terms] OR ("multiple"[All Fields] AND "sclerosis"[All Fields]) OR "multiple sclerosis"[All Fields] OR ("disseminated"[All Fields] AND "sclerosis"[All Fields]) OR "disseminated sclerosis"[All Fields]) OR ("ms"[Journal] OR "med sci paris"[Journal] OR "ms"[All Fields])) AND ("multiple sclerosis"[MeSH Terms] OR ("multiple"[All Fields] AND "sclerosis"[All Fields]) OR "multiple sclerosis"[All Fields])) OR ("multiple sclerosis"[MeSH Terms] OR ("multiple"[All Fields] AND "sclerosis"[All Fields]) OR "multiple sclerosis"[All Fields] OR ("multiple"[All Fields] AND "sclerosis"[All Fields] AND "acute"[All Fields] AND "fulminating"[All Fields]) OR "multiple	11	1:53:17	2026/1/6

PEDro
Physiotherapy Evidence Database

Home | Display Selected Records | New Search (Simple) | Continue Searching (Advanced) | New Search (Advanced) | Search Help

Search Results

Click on a title to view details of that record. If your search has returned many records you may need to move to the next page (at the top or bottom of the list of records). To display a list of records from one or a series of searches, click on *Select* and then *Display Selected Records*

Found 2 records

Title	Method	Score (/10)	Select Record
Do proprioceptive neuromuscular facilitation techniques improve respiratory parameters and swallowing in people with multiple sclerosis: a randomized-controlled study [with consumer summary]	clinical trial	5/10	Select
Effect of expiratory muscle strength training on voice and speech: an exploratory study in persons with Parkinson's disease or multiple sclerosis	clinical trial	5/10	Select

Web of Science Search Strategy (v0.1)

Database: Web of Science Core Collection

Entitlements:

- WOS.IC: 1993 to 2026
- WOS.CCR: 1985 to 2026
- WOS.SCI: 2013 to 2026

Searches:

Search: (((Exercise, Breathing OR Respiratory Muscle Training OR Muscle Training, Respiratory OR Training, Respiratory Muscle) AND (Breathing Exercises)) AND (Sclerosis, Multiple OR Sclerosis, Disseminated OR Disseminated Sclerosis OR MS (Multiple Sclerosis) OR Multiple Sclerosis, Acute Fulminating)) AND (Multiple Sclerosis) (All Fields) and 2025 or 2024 (Publication Years) Date Run: Wed Jan 07 2026 01:17:04 GMT+0800 (中国标准时间) Results: 9

Search: (((Exercise, Breathing OR Respiratory Muscle Training OR Muscle Training, Respiratory OR Training, Respiratory Muscle) AND (Breathing Exercises)) AND (Sclerosis, Multiple OR Sclerosis, Disseminated OR Disseminated Sclerosis OR MS (Multiple Sclerosis) OR Multiple Sclerosis, Acute Fulminating)) AND (Multiple Sclerosis) (All Fields)
Date Run: Wed Jan 07 2026 01:16:58 GMT+0800 (中国标准时间) Results: 50

Filter your results

Date

Publication date

The last 3 months

The last 6 months

The last 9 months

The last year

The last 2 years

Custom Range:

01/03/2024 to dd/mm/yyyy

Apply Clear

Cochrane Reviews	Cochrane Protocols	Trials	Editorials	Special Collections	Clinical Answers
2	0	96	0	0	0

Date: Custom date range ×

0 Cochrane Reviews matching **(((Exercise, Breathing OR Respiratory Muscle Training OR Muscle Training, Respiratory OR Training, Respiratory Muscle) AND (Breathing Exercises)) AND (Sclerosis, Multiple OR Sclerosis, Disseminated OR Disseminated Sclerosis OR MS (Multiple Sclerosis) OR Multiple Sclerosis, Acute Fulminating)) AND (Multiple Sclerosis) in Title Abstract Keyword - (Word variations have been searched)**

Did you mean: *exerciser* | *exercize* | *exorcise*

Cochrane Database of Systematic Reviews

Issue 1 of 12, January 2026

Select all (0) Export selected citation(s) Show all previews

Order by Relevancy

Results per page 25
