# Supplementary material for: Improving respiratory muscle strength in patients with multiple sclerosis through respiratory muscle training: a systematic review and meta-analysis
Source: PeerJ. 2026 Apr 9;14:e20876. doi: 10.7717/peerj.20876 (PMC13070315; doi:10.7717/peerj.20876)
Supplement: Supplemental Information 4 [file peerj-14-20876-s004.docx]

The rationale for conducting the meta-analysis

Multiple sclerosis has a high incidence of this degenerative disease and can occur at any age. It has a serious impact on family and society. At the same time, respiratory dysfunction in multiple sclerosis is also an important cause of death in this population. Therefore, we made a special summary of respiratory dysfunction in respiratory muscle training, hoping to find relevant methods to reduce the death caused by respiratory dysfunction.

The contribution that it makes to knowledge in light of previously published related reports, including other meta-analyses and systematic reviews.

First of all, we specialize in breathing training for multiple sclerosis, and the study of multiple sclerosis goes even further.

Second, the articles we included have a large time span of the included literature, which has a summary of the research of various eras.

Thirdly, we have added FEV1/FVC as our outcome indicator.

Fourth, our meta-analysis had no other outcome measures and focused more on respiratory muscle strength.

Fifth, we have analyzed how various outcome indicators improve the effect of respiratory muscle training, in order to better understand the mechanism of respiratory muscle training to improve multiple sclerosis.

Sixth, all of the trials we included were randomized controlled trials
